# Advances in miR-132-Based Biomarker and Therapeutic Potential in the Cardiovascular System

**DOI:** 10.3389/fphar.2021.751487

**Published:** 2021-11-02

**Authors:** Kaizu Xu, Chungui Chen, Ying Wu, Meifang Wu, Liming Lin

**Affiliations:** ^1^ Department of Cardiology, Affiliated Hospital of Putian University, The Third School of Clinical Medicine, Southern Medical University, Putian, China; ^2^ Department of Radiology, Affiliated Hospital of Putian University, The Third School of Clinical Medicine, Southern Medical University, Putian, China

**Keywords:** miR-132, Biomarker (BM), therapeutic potential, CDR132L, heart failure

## Abstract

Atherosclerotic cardiovascular disease and subsequent heart failure threaten global health and impose a huge economic burden on society. MicroRNA-132 (miR-132), a regulatory RNA ubiquitously expressed in the cardiovascular system, is up-or down-regulated in the plasma under various cardiac conditions and may serve as a potential diagnostic or prognostic biomarker. More importantly, miR-132 in the myocardium has been demonstrated to be a master regulator in many pathological processes of ischemic or nonischemic heart failure in the past decade, such as myocardial hypertrophy, fibrosis, apoptosis, angiogenesis, calcium handling, neuroendocrine activation, and oxidative stress, through downregulating target mRNA expression. Preclinical and clinical phase 1b studies have suggested antisense oligonucleotide targeting miR-132 may be a potential therapeutic approach for ischemic or nonischemic heart failure in the future. This review aims to summarize recent advances in the physiological and pathological functions of miR-132 and its possible diagnostic and therapeutic potential in cardiovascular disease.

## Introduction

With the aging of the population and improved survival of atherosclerotic cardiovascular disease (CVD), the prevalence of heart failure (HF) is increasing worldwide, imposing a huge economic burden on society (Cook et al., 2014; Virani et al., 2020). Despite current advances in drug and device treatment for chronic HF, especially for HF with reduced ejection fraction, the risk of death or readmission for HF within 1 year remains as high as 15% (Crespo-Leiro et al., 2016). Therefore, it is of great clinical relevance to find novel diagnostic and prognostic biomarkers for early diagnosis and risk stratification and new therapeutic drugs for improving the long-term prognosis of HF.

MicroRNAs (miRNAs), first discovered in the 1990s (Lee et al., 1993), are small non-coding RNAs of 18–25 nucleotides that post-transcriptionally regulate gene expression through binding to the 3'untranslated region (UTR) of their target messenger RNAs (mRNAs), resulting in mRNAs degradation and/or translational repression (He and Hannon, 2004; van Rooij, 2011; Bernardo et al., 2012). To date, about 2,300 miRNAs have been identified in the human genome, which are critically involved in biological development, cell differentiation, apoptosis, and many other physiological and pathological processes by regulating up to 60% of human genes at the post-transcriptional level (Ambros, 2004; Latronico and Condorelli, 2009; Krol et al., 2010). Mounting evidence has suggested that many specific miRNAs are up-or down-regulated in the failing human heart (Thum et al., 2007). Among them, miR-132 is well documented to be abnormally expressed under various cardiac stresses and play a pivotal role in regulating the pathological process of hypertrophy, fibrosis, apoptosis, and angiogenesis, which are implicated in the development and progression of ischemic heart failure, thus conferring miR-132 a potential diagnostic biomarker and therapeutic target for ischemic cardiovascular disease.

In the present review, we will discuss the available evidence for the use of miR-132 as diagnostic and prognostic biomarkers for cardiovascular diseases. Next, we will discuss the possible effects and mechanisms of action of aberrant miR-132 expression in the cardiovascular system (e.g., cardiomyocytes, cardiac fibroblasts, endothelial, and vascular smooth muscle cells). Finally, we will summarize the current knowledge and future challenges about antisense oligonucleotide inhibitors of miR-132 as promising therapeutic drugs for heart failure.

## MiR-132 Biogenesis and Regulation

MiR-132 arises from the highly conserved miR-132/212 gene cluster arrayed in tandem on chromosome 17 in humans, with both miRNAs having identical seed regions and possibly sharing some common mRNA targets (Tognini and Pizzorusso, 2012; Wanet et al., 2012). Mature miR-132 mostly follows the canonical pathway of miRNA biogenesis. In brief, miR-132 genes are transcribed by RNA polymerase II from intergenic, intronic, or polycistronic loci to pri-miRNAs and processed in the nucleus by the Drosha–DGCR8 complex to pre-miRNAs of approximately 70 nucleotides. Mirtrons provide an alternative source to form pre-miRNA hairpins. Pre-miRNAs are then exported to the cytoplasm and cleaved by the Dicer–TRBP complex to imperfect miRNA: miRNA* duplexes of 22 nucleotides. One strand of the duplex, the mature miRNA, is loaded into the miRNA-induced silencing complex to exert post-transcriptional negative regulation of target mRNA, while the complementary strand is degraded (Krol et al., 2010; van Rooij and Kauppinen, 2014). The biogenesis of miR-132 is regulated at multiple levels including transcription, Drosha or Dicer processing, RNA editing, argonaute modification and RNA decay (Krol et al., 2010; Ha and Kim, 2014). It has been demonstrated that the transcription of miR-132/212 itself is positively and negatively controlled by cAMP-response element binding (CREB) protein and repressor element 1 silencing transcription factor, respectively (Vo et al., 2005; Conaco et al., 2006; Wanet et al., 2012). Judit Remenyi, et al. further disclosed that the transcription of the miR-132/212 miRNA cluster is also strongly regulated by extracellular-signal-regulated kinase 1/2 (ERK1/2) signaling, in part through the downstream mitogen and stress-activated kinase and the phosphorylation of CREB (Remenyi et al., 2010).

## MiR-132-Based Biomarker Potential in Cardiovascular Disease

The serum levels of miRNAs are highly stable, reproducible, and resistant to harsh conditions such as boiling, low/high pH, extended storage, and freeze-thaw cycles for association with microparticles, RNA-binding protein, or high-density lipoproteins, (Chen et al., 2008; Cortez et al., 2011; Creemers et al., 2012). Besides, the medicines commonly used in the cardiovascular system, including heparin, angiotensin-converting enzyme inhibitors, beta-blockers, nitrates, statins, aspirin, clopidogrel, and n-3 polyunsaturated fatty acids, have been proven not to affect plasma levels of miR-132 (Masson et al., 2018; Li et al., 2019). The above characteristics of miRNAs or miR-132 make serum miR-132 a potential biomarker for disease diagnosis and risk assessment (Table 1).

**TABLE 1:** Circulating miR-132 as potential diagnostic and prognostic biomarkers in cardiovascular disease.

Disease	Study design	Source	Change in expression vs. controls	Clinical application	References
AMI	35 AMI vs. 55 healthy controls	Plasma	↓	Diagnosis	Li et al. (2019)
UAP	10 UAP vs. 10 non-coronary chest pain vs. 10 healthy controls	Serum	↓	Diagnosis	Zeller et al. (2014)
HF	65 HF with LVEF ≤ 45% vs. 62 healthy controls	Plasma	↓	NA	Liu et al. (2018a)
DM without CVD	Patients with different duration of DM (1–5, 6–10, 11–15, and >15 years, *n* = 17, 18, 16, and 17, respectively) vs. age- -matched non-DM	Plasma	↓	Identify diabetic cardiac microangiopathy	Rawal et al. (2017)
CAD	1,112 CAD including 430 ACS and 682 SAP, 4 years follow-up	Serum	NA	Higher miR-132 levels predict CV death in ACS patients	Karakas et al. (2017)
CHF	953 symptomatic CHF from GISSI-HF trial, 46.2 months follow-up	Plasma	NA	Higher miR-132 levels were associated with severe HF symptom, but predicted lower risk of HF readmission	Panico and Condorelli (2018)

AMI, acute myocardial infarction; UAP, unstable angina pectoris; HF, heart failure; NA, not applicable; DM, diabetes mellitus; CVD, cardiovascular disease; CAD, coronary artery disease; ACS, acute coronary syndrome; SAP, stable angina pectoris; CV, cardiovascular; CHF, chronic heart failure.

### Diagnostic Biomarker Potential

Recently, specific expression patterns of serum miR-132 have been documented associated with various cardiovascular diseases. Li, et al. evaluated the dynamic changes in plasma levels of miRNAs and cardiac troponin I (cTnI) of 35 acute myocardial infarction (AMI) patients and 55 matched controls, and found that the circulating level of miR-132-5p was maintained at a low level during the early phase of AMI and negatively correlated with cTnI. Receiver operating characteristic analysis suggested that miR-132-5p may serve as a novel promising diagnostic biomarker for the early diagnosis of AMI (Li et al., 2019). Unlike the diagnosis of AMI distinctly relying on cTnI measurement, early diagnosis of unstable angina pectoris (UAP) remains a major clinical challenge for no available biomarkers providing clinically useful information. Zeller et al. identified eight significantly lower miRNAs, including miR-132, in UAP patients (*n* = 10) than in non-coronary chest pain patients (*n* = 10) and healthy controls (*n* = 10), which facilitate the early diagnosis of UAP. A panel of three miRNAs (miR-132, -150, and -186) showed the highest discriminatory power, with an area under the receiver-operating characteristic curve (AUC) of 0.91 (95% confidence interval: 0.84–0.98), compared with high-sensitivity assayed troponin I (hsTnI) and a model including hsTnI, B-type natriuretic peptide, C-reactive protein, and cystatin C (4-marker combination) (AUC of 0.57 and 0.63, respectively) (Zeller et al., 2014). Liu et al. found that the plasma levels of miR-132 in HF patients with left ventricular ejection fraction less than 45% (*n* = 65) were downregulated compared with healthy controls (*n* = 62) (Liu et al., 2018a). Besides, studies also showed aberrant circulating miR-132 levels in patients without overt cardiovascular disease. Rawal, et al. demonstrated that the miR-132 levels in the plasma and myocardium of diabetes patients without any known history of cardiovascular disease were downregulated compared to healthy subjects, and associated with decreased capillaries and arterioles and increased endothelial cell apoptosis, which is a hallmark of microangiopathy. Thus, monitoring the circulating levels of miR-132 could potentially identify those individuals with preclinical diabetic cardiac microangiopathy (Rawal et al., 2017). More recently, Šatrauskienė et al. identified a cluster of four miRNAs including miR-132, miR-1, miR-122, and miR-133 related to subclinical atherosclerosis in patients with metabolic syndrome (n = 182), suggesting a more substantial diagnostic or prognostic value of combined miRNAs than any single miRNA (Šatrauskienė et al., 2021). The above-mentioned studies suggest that a reduction in plasma miR-132 levels, either singly used or combined with other miRNAs, may have an additive diagnostic value for patients with diabetic cardiac microangiopathy, unstable angina, myocardial infarction, and heart failure. However, the sample size of these studies was mostly small, and their conclusions still need to be further validated by larger clinical studies in the future.

### Prognostic Biomarker Potential

Cardiovascular death risk stratification for patients with coronary artery disease is helpful to guide intensified secondary preventive therapies. Karakas et al. first assessed the association of circulating miRNAs levels with secondary adverse cardiovascular events in a cohort of 1,112 patients with documented coronary artery disease, including 430 patients with acute coronary syndrome and 682 patients with stable angina pectoris. During a median follow-up of 4.0 years, Cox regression analyses adjusted for age and gender indicated that miR-132 precisely predicted cardiovascular death (HR 2.85 per 1 SD increase, *p* = 0.022) in patients with acute coronary syndrome. The C-statistics showed excellent values for prediction of cardiovascular death (AUC for miR-132: 0.737) (Karakas et al., 2017). Instead, another study by Masson et al. drew the opposite results. The authors retrospectively analyzed the circulating levels of miR-132 in 953 patients with chronic, symptomatic heart failure from the GISSI-Heart Failure trial and showed that higher plasma levels of miR-132 were independently associated with increased HF severity (NYHA class and ischaemic etiology), but consistently predicted lower rates of fatal (all-cause or cardiovascular death) or non-fatal events (hospitalization for cardiovascular or HF reasons). After extensive adjustment for demographic, clinical, and echocardiographic risk factors and baseline N-terminal brain natriuretic peptide precursor (NT-proBNP) concentrations, miR-132 remained associated only with HF hospitalizations (HR 0.79, 95% confidence interval 0.66–0.95, *p* = 0.01). Of note, The association was observed in patients with HF of ischaemic etiology but not in those with HF of non-ischaemic etiology (*P* for heterogeneity 0.08). Besides, miR-132 improved risk prediction beyond traditional risk factors for HF hospitalization with the continuous net reclassification index of 0.205 (*p* = 0.001) (Masson et al., 2018; Panico and Condorelli, 2018). Taken together, these results suggest that the translation of circulating miR-132 into clinical prognostic biomarkers may be hampered by lack of consistency or restricted to certain subgroups of patients, such as acute coronary syndrome and ischemic heart failure.

## Physiological and Pathological Roles of miR-132 in Cardiovascular Disease

Through reviewing the existing literature, we've come to the conclusion that miR-132 may play a crucial role in regulating pathophysiological processes of cardiovascular disease, including myocardial hypertrophy, autophagy, fibrosis, apoptosis, angiogenesis, calcium handling, neuroendocrine activation, oxidative stress, and endothelial and vascular smooth muscle cell biology (Table 2).

**TABLE 2:** MiR-132 targets and functions in pathophysiologic process of cardiovascular disease.

Functions	Injury model or mode of action	Upregulation/downregulation	Target molecule	Target pathway	References
Pro-hypertrophy and anti-autophagy	*In vivo* AngII, PE/ISO, and TCA	Upregulation	FoxO3	Calcineurin/NAFT↑	Ucar et al. (2012)
					Eskildsen et al. (2013)
Profibrosis	*In vitro* AngII	Upregulation	MMP-9, FoxO3	NA	Jiang et al. (2013)
					Schimmel et al. (2021)
Antifibrosis	*In vivo* MI, *in vitro* AngII	Upregulation	PTEN	PI3K/Akt↓	Wang et al. (2020)
	*In vivo* DCM	Downregulation	PTEN	PI3K/Akt↑	Zhang et al. (2019)
Antiapoptosis	*In vitro* miR-212/132-Transgenic H9c2	Upregulation	FoxO3	PI3K/Akt↑	Ucar et al. (2012)
	*In vivo* MI	Downregulation	NA	Interleukin-1β↑	Zhao et al. (2020)
Proangiogenesis	*In vivo* MI and hind-limb ischaemia	Upregulation	p120RasGAP, Spred1	Ras-MAPK↑	Lei et al. (2015)
					Katare et al. (2011)
Impaired calcium handling	End-stage heart failure patients of different etiologies	Upregulation	SERCA2	NA	Lei et al. (2021)
	*In vivo* I/R injury	Upregulation	NCX1	NA	Hong et al. (2015)
Interaction with AVP synthesis	Intravenous antagomir-132	Downregulation	MeCP2	AVP synthesis↓	Bijkerk et al. (2018)
Redox regulation	*In vivo* and *in vitro* I/R injury	Upregulation	SIRT1	PGC-1α/NRF2↓	Zhou et al. (2020)
Induction of a Pro‐inflammatory Phenotype in EC	*In vitro* TNF-α-treated EC	Upregulation	SIRT1	SREBP-1c Metabolic Pathway↓	Zhang et al. (2014)
	*In vitro* ox-LDL-treated EC	Upregulation	MGP	JNK↑	Fu et al. (2018)
				NF-κB↑	
Modulation of VSMC behavior	*In vivo* catheter injury	Upregulation	Lrrfip1	Erk1/2 phosphorylation↑	Choe et al. (2013)
	*In vivo* and *in vitro* DM	Upregulation	E2F5	NA	Xu et al. (2019)
	*In vivo* and *in vitro* AngII	Upregulation	PTEN	MCP-1↑	Jin et al. (2012)

AngII, angiotensin; PE/ISO, phenylephrine/isoprenaline; TAC, transaortic constriction; FoxO3, forkhead box protein O3; NAFT, nuclear factor of activated T-cells; MMP-9, matrix metalloproteinase-9; PTEN, phosphatase and tensin homolog; PI3K, phosphateidylinositol3-kinase; Akt, protein kinase B; DCM, dilated cardiomyopathy; TG, transgenic; IL-1β, interleukin-1β; p120RasGAP, p120 Ras GTPase-activating protein; Spred1, Sprouty‐related Ena/VASP homology‐1 domain‐containing protein1; MAPK, mitogen-activated protein kinase; SERCA2, sarcoplasmic reticulum Ca(2+)-ATPase; I/R, ischemia/reperfusion; NCX1, Na(+)-Ca(2+) exchanger 1; MeCP2, methyl-CpG-binding protein 2; PGC-1α, proliferator-activated receptor-gamma coactivator-1α; NRF2, nuclear factor erythroid 2-related factor 2; EC, endothelial; TNF-α, tumor necrosis factor-α; SIRT1, silent information regulator 1; SREBP-1c, sterol regulatory element binding protein-1c; ox-LDL, oxidized low-density lipoprotein; MGP, matrix Gla protein; JNK, c-Jun N-terminal kinase; NF-κB, nuclear factor Kappa B; VSMC, Vascular smooth muscle cell; LRRFIP1, leucine-rich repeat (in Flightless 1) interacting protein-1; Erk1/2, extracellular-signal-regulated kinase 1/2; DM, diabetes millitus; E2F5, E2F transcription factor 5; MCP-1, monocyte chemotaxis protein-1.

### Pro-Hypertrophic and Anti-Autophagic Properties

Pathological cardiac hypertrophy is a hallmark characteristic of heart failure of different etiology. Ucar et al. first reported that both miR-132 and miR-212 were upregulated in cardiomyocytes upon different hypertrophic stimuli both *in vitro* and *in vivo*, such as angiotensin II (AngII), insulin-like growth factor-1, phenylephrine/isoprenaline, and fetal calf serum, and were independently sufficient to induce hypertrophy. Cardiomyocyte-specific overexpression of miR-132/212 leads to pathological cardiac hypertrophy, heart failure, and death in mice. Conversely, miR-132/212-deficient mice or pharmacological inhibition by antagomiR against miR-132/212 rescued pressure overload-induced hypertrophy and prevented heart failure (Ucar et al., 2012). Mechanistically, it was proved that the miR-132/212 family regulates both cardiac hypertrophy and cardiomyocyte autophagy by translationally repressing forkhead box protein O3(FoxO3), a powerful anti-hypertrophic and pro-autophagic transcription factor in cardiomyocytes (Ni et al., 2006; Sengupta et al., 2009; Ronnebaum and Patterson, 2010), leading to hyperactivation of pro-hypertrophic calcineurin/nuclear factor of activated T-cells signaling and impaired autophagic response upon starvation. In line with these findings, Eskildsen et al. demonstrated that the expression of miR-132 was significantly increased in the heart, aortic wall, and kidney, as well as in the plasma of rats with hypertension and cardiac hypertrophy induced by 10 days of AngII infusion (Eskildsen et al., 2013). Narasimhan et al. further revealed that the increased cardiomyocyte expression of miR-132 induced by isoproterenol was related to increased phosphorylation of CREB through activation of the mitogen-activated protein kinase (MAPK)/ERK pathway (Narasimhan et al., 2018).

### Profibrotic Potential

Cardiac fibrosis, characterized by the deposition of excessive extracellular matrix mainly derived from fibroblasts, leads to stiffness of the heart and compromised heart contractility. It is well documented that miR-132/212 functions as master signaling switches to fine-tune the AngII actions in cardiac fibroblasts (CFs). With the global array analysis of AngII-induced miRNA expression, Jeppesen et al. identified five miRNAs, including the miR-132/212 family in primary cultures of CFs that were upregulated by AngII through activation of Gαq/ERK1/2-dependent signaling (Jeppesen et al., 2011). Eskildsen et al. further undertook a detailed analysis of miR-132/212 molecular targets to unravel the role of miR-132 and miR-212 in AngII signaling networks in CFs and found that miR-132/212 overexpression increased fibroblast cell size and affected several hundred genes expression, including a wide panel of receptors, signaling molecules and transcription factors (Eskildsen et al., 2015). Jiang et al. revealed that the levels of miR-132 in CFs were upregulated by AngII and identified matrix metalloproteinase-9 as the target of miR-132 (Jiang et al., 2013). Recently, Schimmel et al. have further confirmed the profibrotic nature of miR-132 through enhancing proliferation and migration activity of primary human cardiac fibroblasts, which was possibly attributed to autophagy repression through targeting FoxO3 (Schimmel et al., 2021). However, there also exist some other studies indicating an opposite view that miR-132 levels are down-regulated in the heart of heart failure rats and Ang-II treated CFs and upregulation of miR-132 exerts inhibitory effects on cardiac fibrosis in MI-induced heart failure rats, doxorubicin-induced dilated cardiomyopathy rat, and canine model of atrial fibrillation (Qiao et al., 2017; Zhang et al., 2019; Wang et al., 2020).

### Prosurvival Action on Cardiomyocytes

Apoptosis in response to cardiac stress, such as myocardial infarction, contributes to an irreversible loss of cardiomyocytes and subsequent adverse remodeling. It is well demonstrated that miR-132/212 plays an anti-apoptotic role by activating the phosphatidylinositol-3 kinase/protein kinase B pathway in cardiomyocytes (Ucar et al., 2012). Overexpression of miR-132 in cardiomyocytes *in vitro* contributes to enhanced resistance to hypoxia, hydrogen peroxide, and hypoxia and glucose deprivation-induced cell death (Liu et al., 2018a; Lei et al., 2020a; Zhang et al., 2020). Besides, *in vivo* studies have shown that miR-132 was downregulated in cardiomyocytes from MI rats compared to sham-operated rats, and overexpression of miR-132 mitigated cardiomyocyte apoptosis and myocardial remodeling, and this effect may be achieved in part through inhibition of interleukin-1β (Zhao et al., 2020). Chen et al. also showed that miR-132 gradually decreased within 7 days post-MI, and the infarct size in miR-132 knockout (KO) mice was larger than that in wild-type mice at postoperative day 14 and day 28, and the cardiac function was worse. MiR-132 mimics at a dose of 16 mg/kg improved cardiac function and reduced infarct size in mice 28 days after MI modeling (Chen et al., 2019). However, Lei et al. reported that the expression of miR-132 initially increased at 12 h post-MI, then decreased at 24 h, but increased nonsignificantly again in later phases within 1 month post-MI. Although miR-132 loss enhanced cardiac contractile function in mice with MI, it also attenuated cardiomyocytes survival and angiogenesis, ultimately not improving overall cardiac performance or fibrosis remodeling 4 weeks post-MI compared with wild-type mice (Lei et al., 2020a).

### Angiogenesis Regulation

Angiogenesis is essential for maintaining oxygen and nutrients supplies to the myocardial tissue, and angiogenesis impairment is involved in the pathogenesis of ischemic heart disease. Accumulating evidence suggests that miRNAs play key roles in regulating vascular endothelium response to angiogenic stimuli, serving as a promising therapeutic approach for ischemic heart diseases involving insufficient vasculature (Fish and Srivastava, 2009). MiR-132 is a proangiogenic miRNA that is highly expressed in endothelial cells and in atherosclerotic lesions in ApoE^−/−^ mice (Xiong et al., 2015). Upon angiogenic stimulation, such as hypoxia (Burek et al., 2019) or loss of functional von Hippel–Lindau gene (Lei et al., 2020b), the miR-132 levels are increased and function as an angiogenic switch by targeting p120 Ras GTPase-activating protein (p120RasGAP, also named RASA1) and Spred1 in the endothelium and thereby leading to Ras-MAPK pathway activation and induction of neovascularization (Anand et al., 2010; Lei et al., 2015). A time-course study in type 2 diabetic mouse model revealed that the down-regulation of miR-132 preceded the development of microangiopathy in the diabetic heart, and therapeutic normalization of miR-132 in *ex vivo* diabetic aortic rings and *in vitro* high glucose-treated human umbilical vein endothelial cells restored their angiogenic potential (Rawal et al., 2017). Moreover, it was documented that miR-132/212 KO mice showing impaired arteriogenic responses after ischemia in the hind limbs compared with wild-type mice (Lei et al., 2015), while intracellular delivery of miR-132 via biodegradable nanoparticles improved endothelial graft survival and blood perfusion after ischemic injury (Gomes et al., 2013).

MiR-132 not only regulates the target gene *in situ* but also acts as a paracrine mediator in affecting angiogenesis at distant sites. Katare et al. reported that transplantation of human pericyte progenitor cells exerted proangiogenic and antifibrotic effects in the infarcted heart through secretion of miR-132 and targeted inhibition of p120RasGAP and methyl-CpG-binding protein 2 (MeCP2), respectively, whereas miR-132 KO in pericytes abrogated these beneficial effects (Katare et al., 2011). Exosomes are effective vectors delivering miR-132 efficiently to the tissue of interest to induce therapeutic angiogenesis for ischemic heart disease (Kir et al., 2018; Moghiman et al., 2021). Barile et al. showed that infarcted hearts injected with miR-132 and miR-210-enriched exosomes from human cardiac progenitor cells exhibited less cardiomyocyte apoptosis, enhanced angiogenesis, and improved ejection fraction compared with those injected with control medium (Barile et al., 2014). Ma et al. also confirmed that delivery of miR-132 via mesenchymal stem cell-derived exosomes in the ischemic hearts of mice markedly enhanced the neovascularization in the peri-infarct zone and preserved heart functions (Ma et al., 2018).

In contrast to the above evidence suggesting that miR-132 has a proangiogenic effect, some studies have concluded otherwise that miR-132 has no major effects on angiogenesis or cardiac capillary densities *in vivo* (Ucar et al., 2012; Kumarswamy et al., 2014), and even in a pressure overload-induced porcine cardiomyopathy model, antagomiR targeting miR-132 improves capillary density (Hinkel et al., 2021). Therefore, there is still no consistent conclusion on the effect of miR-132 on angiogenesis, which needs to be further clarified in future studies.

### Impaired Calcium Handling

Cyclic changes of intracellular calcium concentration are involved in regulating the excitation-contraction coupling of cardiomyocytes (Bers, 2002). It is well known that cardiac sarcoplasmic reticulum Ca(2+)-ATPase (SERCA2) plays a crucial role in modulating cardiac contraction and relaxation by regulating intracellular calcium processing, and attenuated SERCA2 expression or activity leads to impaired calcium handling associated with contractile dysfunction and heart failure progression (Frank et al., 2003). Wahlquist et al. first documented that miR-132/212 can suppress green fluorescent protein expression in the SERCA2 3 ‘-UTR reporter, indicating miR-132/212 may be a regulator of SERCA2 (Wahlquist et al., 2014). Later, Foinquinos et al. demonstrated that overexpression of miR-132 in cardiomyocytes compromised contractile kinetics, which could be normalized by antimiR-132 treatment by, at least in part, restoring SERCA2 expression (Foinquinos et al., 2020). Lei et al. showed that miR-132/212 overexpression prolongs calcium decay in isolated neonatal rat cardiomyocytes, whereas cardiomyocytes isolated from miR-132/212 KO mice display enhanced contractility in comparison to wild type controls. The authors also found upregulation of miR-132/212 and reduced SERCA2 protein expression in end-stage heart failure patients of different etiologies, including dilated cardiomyopathy, hypertrophic cardiomyopathy, and ischemic cardiomyopathy (Lei et al., 2021). Besides, it was also suggested that elevated miR-132/212 can lower SERCA2 activity indirectly via inhibition of PTEN, which is a direct target of miR-132/212 and loss of function in cardiomyocytes leading to a dramatic decrease in contractility (Crackower et al., 2002; Ruan et al., 2009). Another study by Hong et al. showed that delivery of miR-132 blunted intracellular Ca(2+) overload through targeting the Na(+)-Ca(2+) exchanger 1, protecting cardiomyocytes against hypoxia-induced apoptosis (Hong et al., 2015).

### Interaction With Neuroendocrine Activation

AngII controls blood pressure and adverse ventricular remodeling in the pathological process of heart failure through activation of angiotensin II type 1 receptor (AT1R). It has been reported that AngII upregulates the expression of miR-132 by activating the Gαq/ERK1/2 pathway while AT1R blockers reduce plasma levels of miR-132 in human patients (Eskildsen et al., 2013). On the other hand, miR-132 fine-tunes AngII responsiveness by translationally repressing AT1R expression by directly binding to sequence recognition sites in the coding region of human AT1R mRNA (Elton et al., 2008). In addition, animal studies have shown that angiotensin-converting enzyme inhibitor ramipril for the treatment of acute kidney injury can simultaneously inhibit cardiac hypertrophy, fibrosis, and apoptosis, and these cardioprotective effects are partially related to the attenuated miR-132 expression (Rana et al., 2015). Arginine vasopressin (AVP) has been recognized as an important contributor to heart failure development through water retention, hyponatremia, and arterial vasoconstriction (Iovino et al., 2018). Bijkerk et al. identified miR-132 as the first miRNA maintaining the water and osmotic balance in the body by regulating the hypothalamic AVP gene mRNA expression. Specifically, miR-132 can promote AVP synthesis and release into blood by targeting MeCP2 expression, which acts on renal aquaporin-2 and promotes water reabsorption. MiR-132 silencing by antagomiR in mice causes severe weight loss due to acute diuresis and increased plasma osmolality, alone with reduced AVP production and apical aquaporin-2 expression (Bijkerk et al., 2018). Thus, miR-132 antagomiR may be of therapeutic value in acquired hypervolemic/hyponatremic conditions, such as congestive heart failure.

### Redox Regulation

Nuclear factor erythroid 2-related factor 2 (NRF2), is a basic leucine zipper protein that promotes an array of antioxidant genes and phase II detoxifying enzymes expression by binding to antioxidant response elements, playing an important role in maintaining the normal function of cardiomyocytes and cardiac fibroblasts and preventing maladaptive cardiac remodeling and heart failure (Li et al., 2009; Chen and Maltagliati, 2018). Zhou et al. showed that inhibition of miR-132 activated peroxisome proliferator-activated receptor-gamma coactivator-1α/NRF2 signaling by targeting silent information regulator 1 (SIRT1), leading to inhibition of oxidative stress and the expression of pyrotic related proteins nucleotide-bound oligomeric domain-like receptor proteins 3, caspase-1, and interleukin-1, ultimately ameliorating myocardial ischemia-reperfusion injury (Zhou et al., 2020). Consistently, Hinkel et al. found that antimiR-132 treatment increased myocardial NRF2 expression compared to untreated control in a porcine model of pressure-overload-induced heart failure (Hinkel et al., 2021). However, some studies have suggested the opposite results that overexpression of miRNA-132 inhibited oxidative stress induced by H_2_O_2_ in H9C2 cells (Liu et al., 2018b), improving cell viability and apoptosis *in vitro* and alleviating ischemia/reperfusion-induced AMI *in vivo* (Su et al., 2020).

### Endothelium and Vascular Smooth Muscle Cells Behavior Modulation

Endothelial dysfunction is supposed to be the initial step toward atherosclerosis development (Gimbrone and García-Cardeña, 2016). Recent studies have shown that miR-132 may be involved in the process of atherosclerosis and ischemic heart disease by adversely affecting the biological behavior of vascular endothelium. Zhang et al. showed that miR-132 induces pro-inflammatory processes of vascular endothelial inflammation through negatively regulating the expression of SIRT1. Besides, miR-132 promoted apoptosis of HUVECs induced by tumor necrosis factor-α and inhibited its proliferation, viability, and migration (Zhang et al., 2014). Similarly, Fu et al. documented that miR-132 was upregulated in HUVECs under oxidized low-density lipoprotein treatment, which could further decrease the expression of matrix Gla protein (MGP), resulting in increased migration and adhesion-related molecule release through activation of the c-Jun N-terminal kinase and nuclear factor Kappa B pathways (Fu et al., 2018).

Vascular smooth muscle cells (VSMCs) are essential components of the vascular wall, and their abnormal behaviors contribute to various vascular diseases such as atherosclerosis, restenosis, and hypertension (Bochaton-Piallat and Gabbiani, 2005). MiR-132 is abundantly expressed in VSMCs *in vivo* and regulates the biological behavior of VSMCs in response to various types of stress (Elton et al., 2008). Choe et al. demonstrated that miR-132 was upregulated in the rat carotid artery after catheter injury, facilitating to prevent neointimal hyperplasia by regulating VSMCs proliferation, differentiation, and migration. Transfection of a miR-132 mimic significantly inhibited the proliferation and migration of VSMCs and induced VSMCs differentiation and apoptosis through downregulation of the expression of target LRRFIP1 and phosphorylation of ERK1/2 (Choe et al., 2013). Consistently, Xu et al. reported that the expression of miR-132 was significantly decreased and E2F transcription factor 5 (E2F5) upregulated in high glucose-treated VSMCs or those obtained from diabetic rats, Upregulation of miR-132 significantly inhibited the proliferation and migration of diabetic rat or high-glucose-treated VSMCs by targeting E2F5 (Xu et al., 2019). Other studies have suggested that miR-132 may promote a phenotypic switch of VSMCs that is conducive to atherosclerosis. Wen Jin, et al. analyzed the miRNAs profiles regulated by Ang II in VSMCs by using a small RNA sequencing method and documented that the miR-132/212 cluster is upregulated by Ang II in a time- and dose-dependent manner, resulting in increased monocyte chemotaxis protein-1 (MCP-1) expression at least in part through suppression of phosphatase and tensin homolog (PTEN) in rat VSMCs. Notably, the aorta of Ang II-infused mice showed similar upregulation of miR-132 and MCP-1, supporting an *in vivo* relevance (Jin et al., 2012). Chen et al. reported that over-expression of miR-132 in VSMCs led to an attenuation of cilostazol-induced VSMCs differentiation via inhibiting PTEN expression, indicating the adverse effects of miR-132 on VSMCs differentiation (Chen et al., 2018).

## MiR-132-Based Therapeutic Potential in Cardiovascular Disease

There are two main types of miR-132-based therapeutics, one is to suppress abnormally upregulated miR-132 through miR-132 inhibition, and the other is to restore attenuated miR-132 through miR-132 supplement. For this purpose, many strategies have been developed to manipulate miR-132 activity *in vivo*, including antisense oligonucleotides (ASOs) with different chemical modifications for miR-132 inhibition and double-stranded miR-132 mimics for miR-132 restoration. Besides, numerous miR-132 delivery tools have also been developed and include the use of a cholesterol moiety, miRNA sponges, liposomes, adeno-associated viruses, exosomes, and nanoparticles (Chistiakov et al., 2012; Bernardo et al., 2015).

### Therapeutic Potential of miR-132 Inhibition in Cardiovascular Disease

CDR132L is a first-in-class, optimized, synthetic locked nucleic acid (LNA) antisense oligonucleotide inhibitor of miR-132 (antimiR-132) (Lu and Thum, 2019; Foinquinos et al., 2020). The inclusion of LNA nucleotides in the antisense oligonucleotide increases both stability and thermodynamic strength of duplex formation with complementary target mRNA (Elmén et al., 2008) Foinquinos et al. first tested the efficacy of antimiR-132 in miR-132/212 transgenic mice. The miR-132/212 transgenic mice showed severe left ventricular hypertrophy, decreased ejection fraction and cardiac dilatation. Pharmacologic inhibition of miR-132 by intravenous injection of antimiR-132 reduced the expression level of miR-132 in the myocardium and restored the expression level of FoxO3, eventually reducing cardiac mass and ventricular dilatation while improving ejection fraction (Foinquinos et al., 2020). In a blind, randomized, placebo-controlled study, Batkai et al. administered monthly intravenous CDR132L to chronic heart failure pigs 1 month after myocardial infarction for 3 or 5 months and assessed the efficacy with magnetic resonance imaging (MRI), hemodynamic, and biomarker tests. The study found that CDR132L treatment achieved sufficient tissue exposure to significantly reverse cardiac remodeling, as evidenced by reduced left ventricular end-systolic volume and left atrial volume on MRI scan and attenuated myocardial interstitial fibrosis and cardiomyocyte size assessed by histology, resulting in improved LVEF by 7.96 and 7.14% as measured by MRI after 3 and 5 months of treatment, respectively, as compared with placebo. Besides, CDR132L also ameliorated diastolic function as evidenced by decreased end-diastolic pressure–volume relationship and minimum rate of change of left ventricular pressure determined by hemodynamic assay, and reduced the plasma level of NT-proBNP (Batkai et al., 2021). Recently, Hinkel et al. established a novel preclinical porcine model of nonischemic pressure-overload hypertrophy by placing a reduction stent in the descending thoracic aorta and assessed the efficacy of intracoronary administration of antimiR-132 at the time of stent implantation and 4 weeks later, finding that antimiR-132 reduces cardiomyocyte cross-sectional area, retards fibrosis, and improves capillary density and LV ejection fraction (antimiR-132 vs. untreated control, 48.9 ± 1.0% vs. 36.1 ± 1.7%, respectively at the 8-week time point (Hinkel et al., 2021; Robson, 2021). The results of Hinkel et al. suggest that CDR132L has potential clinical application in hypertrophic heart disease caused by non-ischemic etiologies, such as aortic stenosis or systemic hypertension (Condorelli and Ferrante, 2021). A first-in-human Phase 1b randomized, double-blind, placebo-controlled clinical trial was conducted to evaluate the safety, pharmacokinetic characteristics, and efficacy of CDR132L in patients with chronic ischemic heart failure receiving standard treatment. A total of 28 patients with LVEF of 30–50% or NT-proBNP ≥ 125 ng/L, age of 30–80 years old, and BMI of 18–28 kg/m^2^ were included in this study and randomly assigned at 5:2 to CDR132L group (20 cases in total, five patients in each cohort receiving 0.32, 1, 3, and 10 mg/kg body weight of CDR132L, respectively) and placebo group (eight cases, 0.9% saline). After a 6-week screening period, subjects were given two doses of CDR132L or placebo by intravenous injection on day 1 and day 28, respectively, and the trial ended on day 112. In this study, CDR132L was safe and well-tolerated. CDR132L treatment resulted in a sustained and sharp decrease in plasma miR-132 levels in a dose-dependent manner. For patients with ischemic chronic heart failure receiving standard treatment, CDR132L can further reduce the median level of NT-proBNP and narrow the QRS wave relative to placebo, and improve nonsignificantly cardiac fibrosis biomarkers as well (Täubel et al., 2021). This is the first human study to target miR-132 and represents a milestone in the field of miRNA therapy for cardiovascular disease. While it is too early to determine whether this strategy will be effective in humans, the study's evidence of the safety and efficacy of CDR132L provides great encouragement for further research in patients with heart failure (Baker and Giacca, 2021; Nicholls, 2021).

### Therapeutic Potential of miR-132 Overexpression in Cardiovascular Disease

Given that the majority of current evidence suggests that miR-132 overexpression is involved in cardiac pathology, most of the miR-132-based therapies mainly focused on miR-132 inhibition, and only a few studies have explored the possible cardiac benefits of miR-132 upregulation. Gupta et al. showed that miR-132 overexpression can prevent cardiac toxicity caused by chemotherapeutic drugs. The authors established a mouse model of doxorubicin-induced cardiotoxicity and found that adeno-associated virus (AAV)9-mediated overexpression of miR-212/132 can counteract doxorubicin-induced cardiotoxicity, increase left ventricular mass and wall thickness, decrease doxorubicin-mediated apoptosis, and ultimately improve ejection fraction, which is partly related to the inhibition of target storage-inducing transmembrane protein 2 (Gupta et al., 2019). Jover et al. first reported that miR-132 was constitutively expressed by adventitial pericytes (APCs) and upregulated following high phosphate stimulation, playing a key role in the human APCs resistance to calcification through downregulating several target genes relevant to osteogenic differentiation. Treatment of swine cardiac valves with APCs-derived conditioned medium conferred them with resistance to high phosphate-induced osteogenesis, with this effect being negated when using the medium of miR-132-silenced APCs (Jover et al., 2021).

## Clinical Perspectives and Future Challenges

### Challenges of Plasma miR-132 Level as a Biomarker

Despite a few small clinical studies have suggested that plasma levels of miR-132 are potential diagnostic or prognostic biomarkers for cardiovascular diseases, several issues need to be addressed before clinical application, such as whether miR-132 levels are affected by food and drugs, and whether they are affected by common comorbidities such as old age, atrial fibrillation, liver or renal insufficiency, and anemia. Does miR-132 provide additional value beyond current traditional risk factors? What is the optimal threshold for diagnosing or indicating a poor prognosis of cardiovascular disease?

### Who Might Benefit From miR-132 Inhibition?

The current evidence supporting miR-132 inhibition as a potential therapeutic approach for heart failure is mainly derived from several animal models with experimental MI and a phase 1b clinical study demonstrating a further reduction in the level of NTpro-BNP in patients with ischemic heart failure receiving standard treatment. Whether miR-132 inhibition can reduce heart failure hospitalization or cardiovascular death in patients with ischemic heart failure and improve cardiac remodeling in patients with non-ischemic heart failure remains to be addressed in future studies.

### What is the Optimal Therapeutic Strategy for miR-132 Inhibition?

Although antimiR-132 targeting miR-132 administered to large animals post-MI has been proven effective in alleviating cardiac remodeling, improving cardiac systolic and diastolic functions, and reducing NT-proBNP (Batkai et al., 2021), knockdown of miR-132/212 has been documented to have no long-term beneficial effect on cardiac function after permanent coronary ligation in mice (Lei et al., 2020a). Besides, a circular miRNA sponge targeting the miR-132/212 family has been recently constructed to effectively attenuate pressure overload-induced cardiac hypertrophy *in vivo* and show greater *in vitro* efficacy than the current gold standard antagomiRs in inhibiting miRNA function (Lavenniah et al., 2020). Thus, the optimal therapeutic strategy for miR-132 silencing in the treatment of myocardial infarction remains unknown and needs to be further clarified.

### Is Systemic Delivery of AntimiR-132 Safe?

Although the short-term safety of systemic administration of antimiR-132 has been preliminarily confirmed in large animal models and clinical phase 1b study (Foinquinos et al., 2020; Täubel et al., 2021), the concern of long-term off-target effects of systemic delivery still needs to be addressed with caution, given the fact that miR-132 is widely expressed and exhibits different functions in different organs or cell types. For example, while inhibiting cardiac hypertrophy, systemic delivery of antimiR-132 may simultaneously increase the risk of neurodegenerative diseases or delay wound healing (Li et al., 2015; El Fatimy et al., 2018). In addition, the high stability of antagomiRs and LNA can also be a double-edged sword, increasing their side effects in other organs (Kwekkeboom et al., 1979). Thus, further studies are required to comprehensively evaluate the long-term safety of systemic delivery of antimiR-132.

### Cell-Specific Delivery of AntimiR-132 May Hold Promise

To circumvent issues of possible off-target effect, high costs, and low efficacy of systemic delivery, cell-specific targeted delivery has become a research hotspot in recent years (Kwekkeboom et al., 1979; Boon and Dimmeler, 2015). Several viral and non-viral vector-based delivery systems, including adenoviral, liposomal, polymer-based nanoparticles, and natural microvesicles/exosomes, have hence been developed to deliver miRNAs inhibitor or mimic specifically and efficiently to the tissue of interest (Kir et al., 2018). It is supposed that the cell-specific delivery of antimiR-132 may likely replace current systemic delivery in the future.

## Conclusion

**FIGURE 1:**
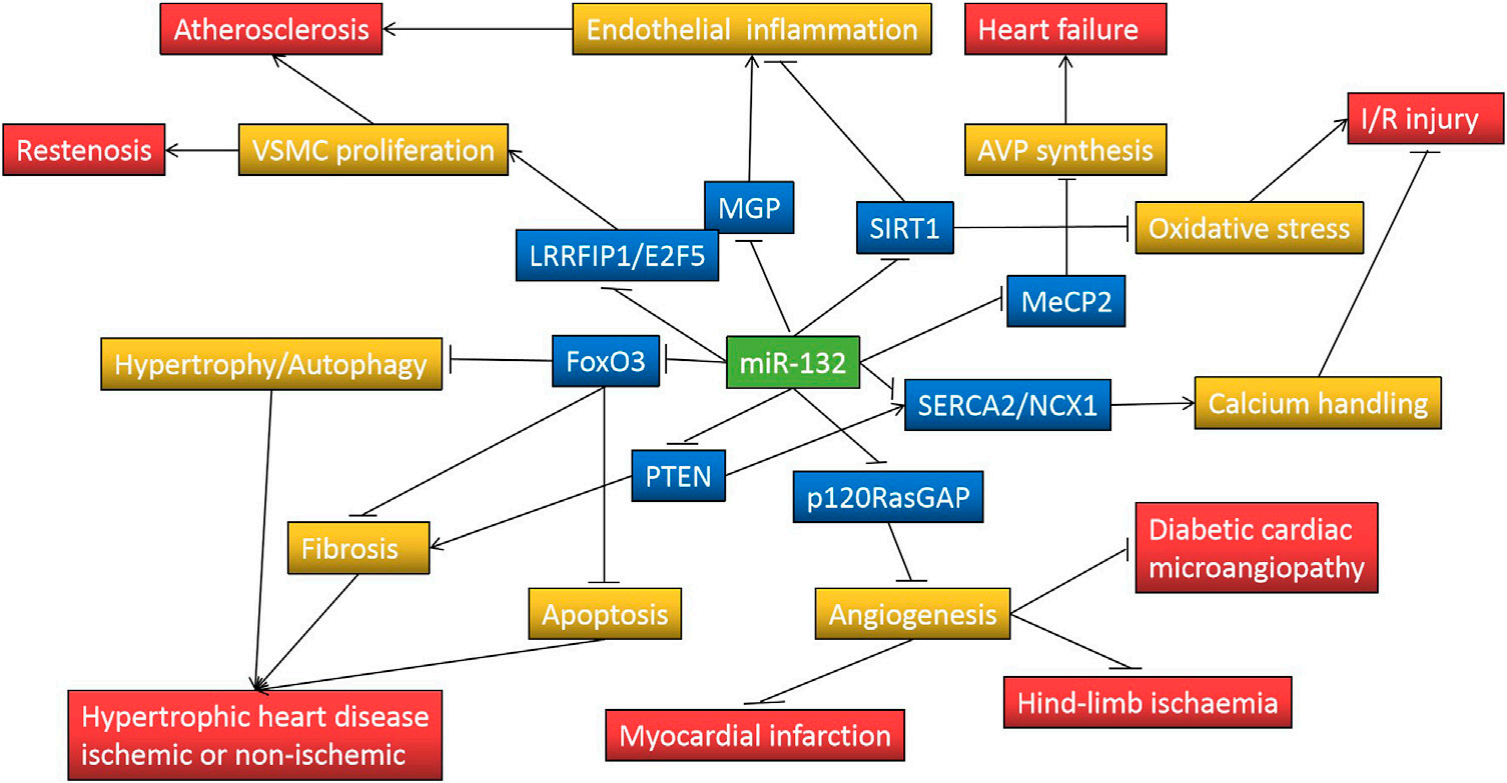
MiR-132 regulates pathological processes involved in cardiovascular disease through repressing target genes expression. Symbol “↑” indicates increase and “⊥” indicates decrease; FoxO3, forkhead box protein O3; PTEN, phosphatase and tensin homolog; p120RasGAP, p120 Ras GTPase-activating protein; SERCA2, sarcoplasmic reticulum Ca(2+)-ATPase; NCX1, Na(+)-Ca(2+) exchanger 1; MeCP2, methyl-CpG-binding protein 2; SIRT1, silent information regulator 1; MGP, matrix Gla protein; LRRFIP1, leucine-rich repeat (in Flightless 1) interacting protein-1; E2F5, E2F transcription factor 5; AVP, arginine vasopressin; VSMC, Vascular smooth muscle cell; I/R, ischemia/reperfusion.

Several clinical studies have suggested that decreased plasma miR-132 levels have additional diagnostic value in acute myocardial infarction, unstable angina, heart failure, and even subclinical atherosclerosis, and may be associated with poor prognosis in patients with heart failure. However, most of these studies have small sample sizes or are retrospective, the reliability of their conclusions needs to be further confirmed by other large prospective studies. In addition, many preclinical studies have documented that the expression of miR-132 in the myocardium is up-regulated under various cardiac stresses and drives some basic pathological processes of heart failure, such as cardiac hypertrophy, fibrosis, and impaired calcium handling, through downregulation of FOXO3A, SERCA2a, PTEN, SIRT1 and other target gene expression, while targeted inhibition of miR-132 by antimiR-132 attenuates cardiac hypertrophy and improves cardiac function (Figure 1). It is encouraging that antimiR-132 has also been demonstrated for the first time in phase 1b clinical trial to further reduce NT-proBNP in patients with ischemic heart failure receiving standard treatment and that the drug is safe and well-tolerated. In the future, more evidence needs to be accumulated on its indications, optimal therapeutic strategy and delivery tool, and long-term safety before translation into clinical practice as a novel anti-heart failure drug.

## References

[B1] Ambros V. (2004). The Functions of Animal microRNAs. Nature 431 (7006), 350–355. 10.1038/nature02871 15372042

[B2] Anand S. Majeti B. K. Acevedo L. M. Murphy E. A. Mukthavaram R. Scheppke L. (2010). MicroRNA-132-Mediated Loss of p120RasGAP Activates the Endothelium to Facilitate Pathological Angiogenesis. Nat. Med. 16 (8), 909–914. 10.1038/nm.2186 20676106 PMC3094020

[B3] Baker A. H. Giacca M. (2021). Antagonism of miRNA in Heart Failure: First Evidence in Human. Eur. Heart J. 42 (2), 189–191. 10.1093/eurheartj/ehaa967 33338200 PMC7816691

[B4] Barile L. Lionetti V. Cervio E. Matteucci M. Gherghiceanu M. Popescu L. M. (2014). Extracellular Vesicles from Human Cardiac Progenitor Cells Inhibit Cardiomyocyte Apoptosis and Improve Cardiac Function after Myocardial Infarction. Cardiovasc. Res. 103 (4), 530–541. 10.1093/cvr/cvu167 25016614

[B5] Batkai S. Genschel C. Viereck J. Rump S. Bär C. Borchert T. (2021). CDR132L Improves Systolic and Diastolic Function in a Large Animal Model of Chronic Heart Failure. Eur. Heart J. 42 (2), 192–201. 10.1093/eurheartj/ehaa791 33089304 PMC7813625

[B6] Bernardo B. C. Charchar F. J. Lin R. C. McMullen J. R. (2012). A microRNA Guide for Clinicians and Basic Scientists: Background and Experimental Techniques. Heart Lung Circ. 21 (3), 131–142. 10.1016/j.hlc.2011.11.002 22154518

[B7] Bernardo B. C. Ooi J. Y. Lin R. C. McMullen J. R. (2015). miRNA Therapeutics: a New Class of Drugs with Potential Therapeutic Applications in the Heart. Future Med. Chem. 7 (13), 1771–1792. 10.4155/fmc.15.107 26399457

[B8] Bers D. M. (2002). Cardiac Excitation-Contraction Coupling. Nature 415 (6868), 198–205. 10.1038/415198a 11805843

[B9] Bijkerk R. Trimpert C. van Solingen C. de Bruin R. G. Florijn B. W. Kooijman S. (2018). MicroRNA-132 Controls Water Homeostasis through Regulating MECP2-Mediated Vasopressin Synthesis. Am. J. Physiol. Ren. Physiol. 315 (4), F1129–f1138. 10.1152/ajprenal.00087.2018 29846108

[B10] Bochaton-Piallat M. L. Gabbiani G. (2005). Modulation of Smooth Muscle Cell Proliferation and Migration: Role of Smooth Muscle Cell Heterogeneity. Handb. Exp. Pharmacol. 170, 645–663. 10.1007/3-540-27661-0_24 16596818

[B11] Boon R. A. Dimmeler S. (2015). MicroRNAs in Myocardial Infarction. Nat. Rev. Cardiol. 12 (3), 135–142. 10.1038/nrcardio.2014.207 25511085

[B12] Burek M. König A. Lang M. Fiedler J. Oerter S. Roewer N. (2019). Hypoxia-Induced MicroRNA-212/132 Alter Blood-Brain Barrier Integrity through Inhibition of Tight Junction-Associated Proteins in Human and Mouse Brain Microvascular Endothelial Cells. Transl. Stroke Res. 10 (6), 672–683. 10.1007/s12975-018-0683-2 30617994 PMC6842347

[B13] Chen L. Wang G. Y. Dong J. H. Cheng X. J. (2019). MicroRNA-132 Improves Myocardial Remodeling after Myocardial Infarction. Eur. Rev. Med. Pharmacol. Sci. 23, 6299–6306. 10.26355/eurrev_201907_18452 31364135

[B14] Chen Q. M. Maltagliati A. J. (2018). Nrf2 at the Heart of Oxidative Stress and Cardiac protection. Physiol. Genomics 50 (2), 77–97. 10.1152/physiolgenomics.00041.2017 29187515 PMC5867612

[B15] Chen W. J. Chen Y. H. Hsu Y. J. Lin K. H. Yeh Y. H. (2018). MicroRNA-132 Targeting PTEN Contributes to Cilostazol-Promoted Vascular Smooth Muscle Cell Differentiation. Atherosclerosis 274, 1–7. 10.1016/j.atherosclerosis.2018.04.030 29738818

[B16] Chen X. Ba Y. Ma L. Cai X. Yin Y. Wang K. (2008). Characterization of microRNAs in Serum: a Novel Class of Biomarkers for Diagnosis of Cancer and Other Diseases. Cell Res. 18 (10), 997–1006. 10.1038/cr.2008.282 18766170

[B17] Chistiakov D. A. Sobenin I. A. Orekhov A. N. (2012). Strategies to Deliver microRNAs as Potential Therapeutics in the Treatment of Cardiovascular Pathology. Drug Deliv. 19 (8), 392–405. 10.3109/10717544.2012.738436 23173580

[B18] Choe N. Kwon J. S. Kim J. R. Eom G. H. Kim Y. Nam K. I. (2013). The microRNA miR-132 Targets Lrrfip1 to Block Vascular Smooth Muscle Cell Proliferation and Neointimal Hyperplasia. Atherosclerosis 229 (2), 348–355. 10.1016/j.atherosclerosis.2013.05.009 23880186

[B19] Conaco C. Otto S. Han J. J. Mandel G. (2006). Reciprocal Actions of REST and a microRNA Promote Neuronal Identity. Proc. Natl. Acad. Sci. U S A. 103 (7), 2422–2427. 10.1073/pnas.0511041103 16461918 PMC1413753

[B20] Condorelli G. Ferrante G. (2021). MicroRNA-132 Inhibition Prevents Myocardial Hypertrophy and Heart Failure in Pigs: Making Sense Out of Antisense. J. Am. Coll. Cardiol. 77 (23), 2936–2938. 10.1016/j.jacc.2021.04.039 34112320

[B21] Cook C. Cole G. Asaria P. Jabbour R. Francis D. P. (2014). The Annual Global Economic burden of Heart Failure. Int. J. Cardiol. 171 (3), 368–376. 10.1016/j.ijcard.2013.12.028 24398230

[B22] Cortez M. A. Bueso-Ramos C. Ferdin J. Lopez-Berestein G. Sood A. K. Calin G. A. (2011). MicroRNAs in Body Fluids-the Mix of Hormones and Biomarkers. Nat. Rev. Clin. Oncol. 8 (8), 467–477. 10.1038/nrclinonc.2011.76 21647195 PMC3423224

[B23] Crackower M. A. Oudit G. Y. Kozieradzki I. Sarao R. Sun H. Sasaki T. (2002). Regulation of Myocardial Contractility and Cell Size by Distinct PI3K-PTEN Signaling Pathways. Cell 110 (6), 737–749. 10.1016/s0092-8674(02)00969-8 12297047

[B24] Creemers E. E. Tijsen A. J. Pinto Y. M. (2012). Circulating microRNAs: Novel Biomarkers and Extracellular Communicators in Cardiovascular Disease? Circ. Res. 110 (3), 483–495. 10.1161/CIRCRESAHA.111.247452 22302755

[B25] Crespo-Leiro M. G. Anker S. D. Maggioni A. P. Coats A. J. Filippatos G. Ruschitzka F. (2016). European Society of Cardiology Heart Failure Long-Term Registry (ESC-HF-LT): 1-year Follow-Up Outcomes and Differences Across Regions. Eur. J. Heart Fail. 18 (6), 613–625. 10.1002/ejhf.566 27324686

[B26] El Fatimy R. Li S. Chen Z. Mushannen T. Gongala S. Wei Z. (2018). MicroRNA-132 Provides Neuroprotection for Tauopathies via Multiple Signaling Pathways. Acta Neuropathol. 136 (4), 537–555. 10.1007/s00401-018-1880-5 29982852 PMC6132948

[B27] Elmén J. Lindow M. Schütz S. Lawrence M. Petri A. Obad S. (2008). LNA-mediated microRNA Silencing in Non-human Primates. Nature 452 (7189), 896–899. 10.1038/nature06783 18368051

[B28] Elton T. S. Kuhn D. E. Malana G. E. Martin M. M. Nuovo G. J. Pleister A. P. (2008). MiR-132 Regulates Angiotensin II Type 1 Receptor Expression through a Protein Coding Region Binding Site. Circulation 118, S513. 10.1161/circ.118.suppl_18.S_513

[B29] Eskildsen T. V. Jeppesen P. L. Schneider M. Nossent A. Y. Sandberg M. B. Hansen P. B. (2013). Angiotensin II Regulates microRNA-132/-212 in Hypertensive Rats and Humans. Int. J. Mol. Sci. 14 (6), 11190–11207. 10.3390/ijms140611190 23712358 PMC3709727

[B30] Eskildsen T. V. Schneider M. Sandberg M. B. Skov V. Brønnum H. Thomassen M. (2015). The microRNA-132/212 Family fine-tunes Multiple Targets in Angiotensin II Signalling in Cardiac Fibroblasts. J. Renin Angiotensin Aldosterone Syst. 16 (4), 1288–1297. 10.1177/1470320314539367 25031299

[B31] Fish J. E. Srivastava D. (2009). MicroRNAs: Opening a New Vein in Angiogenesis Research. Sci. Signal. 2 (52), pe1. 10.1126/scisignal.252pe1 19126861 PMC2680274

[B32] Foinquinos A. Batkai S. Genschel C. Viereck J. Rump S. Gyöngyösi M. (2020). Preclinical Development of a miR-132 Inhibitor for Heart Failure Treatment. Nat. Commun. 11 (1), 633–710. 10.1038/s41467-020-14349-2 32005803 PMC6994493

[B33] Frank K. F. Bölck B. Erdmann E. Schwinger R. H. (2003). Sarcoplasmic Reticulum Ca2+-ATPase Modulates Cardiac Contraction and Relaxation. Cardiovasc. Res. 57 (1), 20–27. 10.1016/s0008-6363(02)00694-6 12504810

[B34] Fu C. Yin D. Nie H. Sun D. (2018). Notoginsenoside R1 Protects HUVEC against Oxidized Low Density Lipoprotein (Ox-LDL)-Induced Atherogenic Response via Down-Regulating miR-132. Cell Physiol. Biochem. 51 (4), 1739–1750. 10.1159/000495677 30504696

[B35] Gimbrone M. A. Jr. García-Cardeña G. (2016). Endothelial Cell Dysfunction and the Pathobiology of Atherosclerosis. Circ. Res. 118 (4), 620–636. 10.1161/CIRCRESAHA.115.306301 26892962 PMC4762052

[B36] Gomes R. S. das Neves R. P. Cochlin L. Lima A. Carvalho R. Korpisalo P. (2013). Efficient Pro-survival/angiogenic miRNA Delivery by an MRI-Detectable Nanomaterial. ACS nano 7 (4), 3362–3372. 10.1021/nn400171w 23451983

[B37] Gupta S. K. Garg A. Avramopoulos P. Engelhardt S. Streckfuss-Bömeke K. Batkai S. (2019). miR-212/132 Cluster Modulation Prevents Doxorubicin-Mediated Atrophy and Cardiotoxicity. Mol. Ther. 27 (1), 17–28. 10.1016/j.ymthe.2018.11.004 30527757 PMC6319305

[B38] Ha M. Kim V. N. (2014). Regulation of microRNA Biogenesis. Nat. Rev. Mol. Cel Biol. 15 (8), 509–524. 10.1038/nrm3838 25027649

[B39] He L. Hannon G. J. (2004). MicroRNAs: Small RNAs with a Big Role in Gene Regulation. Nat. Rev. Genet. 5 (7), 522–531. 10.1038/nrg1379 15211354

[B40] Hinkel R. Batkai S. Bähr A. Bozoglu T. Straub S. Borchert T. (2021). AntimiR-132 Attenuates Myocardial Hypertrophy in an Animal Model of Percutaneous Aortic Constriction. J. Am. Coll. Cardiol. 77 (23), 2923–2935. 10.1016/j.jacc.2021.04.028 34112319

[B41] Hong S. Lee J. Seo H. H. Lee C. Y. Yoo K. J. Kim S. M. (2015). Na(+)-Ca(2+) Exchanger Targeting miR-132 Prevents Apoptosis of Cardiomyocytes under Hypoxic Condition by Suppressing Ca(2+) Overload. Biochem. Biophys. Res. Commun. 460 (4), 931–937. 10.1016/j.bbrc.2015.03.129 25839659

[B42] Iovino M. Iacoviello M. De Pergola G. Licchelli B. Iovino E. Guastamacchia E. (2018). Vasopressin in Heart Failure. Endocr. Metab. Immune Disord. Drug Targets 18 (5), 458–465. 10.2174/1871530318666180212095235 29437026

[B43] Jeppesen P. L. Christensen G. L. Schneider M. Nossent A. Y. Jensen H. B. Andersen D. C. (2011). Angiotensin II Type 1 Receptor Signalling Regulates microRNA Differentially in Cardiac Fibroblasts and Myocytes. Br. J. Pharmacol. 164 (2), 394–404. 10.1111/j.1476-5381.2011.01375.x 21449976 PMC3174419

[B44] Jiang X. Ning Q. Wang J. (2013). Angiotensin II Induced Differentially Expressed microRNAs in Adult Rat Cardiac Fibroblasts. J. Physiol. Sci. 63 (1), 31–38. 10.1007/s12576-012-0230-y 23007623 PMC10717151

[B45] Jin W. Reddy M. A. Chen Z. Putta S. Lanting L. Kato M. (2012). Small RNA Sequencing Reveals microRNAs that Modulate Angiotensin II Effects in Vascular Smooth Muscle Cells. J. Biol. Chem. 287 (19), 15672–15683. 10.1074/jbc.M111.322669 22431733 PMC3346099

[B46] Jover E. Fagnano M. Cathery W. Slater S. Pisanu E. Gu Y. (2021). Human Adventitial Pericytes Provide a Unique Source of Anti-calcific Cells for Cardiac Valve Engineering: Role of microRNA-132-3p. Free Radic. Biol. Med. 165, 137–151. 10.1016/j.freeradbiomed.2021.01.029 33497799

[B47] Karakas M. Schulte C. Appelbaum S. Ojeda F. Lackner K. J. Münzel T. (2017). Circulating microRNAs Strongly Predict Cardiovascular Death in Patients with Coronary Artery Disease-Results from the Large AtheroGene Study. Eur. Heart J. 38 (7), 516–523. 10.1093/eurheartj/ehw250 27357355

[B48] Katare R. Riu F. Mitchell K. Gubernator M. Campagnolo P. Cui Y. (2011). Transplantation of Human Pericyte Progenitor Cells Improves the Repair of Infarcted Heart through Activation of an Angiogenic Program Involving Micro-RNA-132. Circ. Res. 109 (8), 894–906. 10.1161/CIRCRESAHA.111.251546 21868695 PMC3623091

[B49] Kir D. Schnettler E. Modi S. Ramakrishnan S. (2018). Regulation of Angiogenesis by microRNAs in Cardiovascular Diseases. Angiogenesis 21 (4), 699–710. 10.1007/s10456-018-9632-7 29956018

[B50] Krol J. Loedige I. Filipowicz W. (2010). The Widespread Regulation of microRNA Biogenesis, Function and Decay. Nat. Rev. Genet. 11 (9), 597–610. 10.1038/nrg2843 20661255

[B51] Kumarswamy R. Volkmann I. Beermann J. Napp L. C. Jabs O. Bhayadia R. (2014). Vascular Importance of the miR-212/132 Cluster. Eur. Heart J. 35 (45), 3224–3231. 10.1093/eurheartj/ehu344 25217442

[B52] Kwekkeboom R. F. Lei Z. Doevendans P. A. Musters R. J. Sluijter J. P. (1979). Targeted Delivery of miRNA Therapeutics for Cardiovascular Diseases: Opportunities and Challenges. Clin. Sci. 127 (6), 351–365. 10.1042/CS20140005 24895056

[B53] Latronico M. V. Condorelli G. (2009). MicroRNAs and Cardiac Pathology. Nat. Rev. Cardiol. 6 (6), 419–429. 10.1038/nrcardio.2009.56 19434076

[B54] Lavenniah A. Luu T. D. A. Li Y. P. Lim T. B. Jiang J. Ackers-Johnson M. (2020). Engineered Circular RNA Sponges Act as miRNA Inhibitors to Attenuate Pressure Overload-Induced Cardiac Hypertrophy. Mol. Ther. 28 (6), 1506–1517. 10.1016/j.ymthe.2020.04.006 32304667 PMC7264434

[B55] Lee R. C. Feinbaum R. L. Ambros V. (1993). The *C. elegans* Heterochronic Gene Lin-4 Encodes Small RNAs with Antisense Complementarity to Lin-14. Cell 75 (5), 843–854. 10.1016/0092-8674(93)90529-y 8252621

[B56] Lei Z. Fang J. Deddens J. C. Metz C. H. G. van Eeuwijk E. C. M. El Azzouzi H. (2020). Loss of miR-132/212 Has No Long-Term Beneficial Effect on Cardiac Function after Permanent Coronary Occlusion in Mice. Front. Physiol. 11, 590. 10.3389/fphys.2020.00590 32612537 PMC7309700

[B57] Lei Z. Klasson T. D. Brandt M. M. van de Hoek G. Logister I. Cheng C. (2020). Control of Angiogenesis via a VHL/miR-212/132 Axis. Cells 9 (4), 1017. 10.3390/cells9041017 PMC7226144 32325871

[B58] Lei Z. van Mil A. Brandt M. M. Grundmann S. Hoefer I. Smits M. (2015). MicroRNA-132/212 Family Enhances Arteriogenesis after Hindlimb Ischaemia through Modulation of the Ras-MAPK Pathway. J. Cell Mol. Med. 19 (8), 1994–2005. 10.1111/jcmm.12586 25945589 PMC4549050

[B59] Lei Z. Wahlquist C. El Azzouzi H. Deddens J. C. Kuster D. Van Mil A. (2021). miR-132/212 Impairs Cardiomyocytes Contractility in the Failing Heart by Suppressing SERCA2a. Front. Cardiovasc. Med. 8, 138. 10.3389/fcvm.2021.592362 PMC8017124 33816571

[B60] Li D. Wang A. Liu X. Meisgen F. Grünler J. Botusan I. R. (2015). MicroRNA-132 Enhances Transition from Inflammation to Proliferation During Wound Healing. J. Clin. Invest. 125 (8), 3008–3026. 10.1172/JCI79052 26121747 PMC4563743

[B61] Li H. Zhang P. Li F. Yuan G. Wang X. Zhang A. (2019). Plasma miR-22-5p, miR-132-5p, and miR-150-3p are Associated with Acute Myocardial Infarction. Biomed. Res. Int. 2019, 5012648. 10.1155/2019/5012648 31179325 PMC6507259

[B62] Li J. Ichikawa T. Villacorta L. Janicki J. S. Brower G. L. Yamamoto M. (2009). Nrf2 Protects Against Maladaptive Cardiac Responses to Hemodynamic Stress. Arterioscler Thromb. Vasc. Biol. 29 (11), 1843–1850. 10.1161/ATVBAHA.109.189480 19592468 PMC12952473

[B63] Liu X. Tong Z. Chen K. Hu X. Jin H. Hou M. (2018). The Role of miRNA-132 Against Apoptosis and Oxidative Stress in Heart Failure. Biomed. Res. Int. 2018, 3452748. 10.1155/2018/3452748 29682535 PMC5845498

[B64] Liu X. Tong Z. Chen K. (2018). The Role of miRNA-132 against Apoptosis and Oxidative Stress in Heart Failure. Biomed. Res. Int. 2018, 3452748. 10.1155/2018/3452748 29682535 PMC5845498

[B65] Lu D. Thum T. (2019). RNA-based Diagnostic and Therapeutic Strategies for Cardiovascular Disease. Nat. Rev. Cardiol. 16 (11), 661–674. 10.1038/s41569-019-0218-x 31186539

[B66] Ma T. Chen Y. Chen Y. Meng Q. Sun J. Shao L. (2018). MicroRNA-132, Delivered by Mesenchymal Stem Cell-Derived Exosomes, Promote Angiogenesis in Myocardial Infarction. Stem Cell Int. 2018, 3290372. 10.1155/2018/3290372 PMC6151206 30271437

[B67] Masson S. Batkai S. Beermann J. Bär C. Pfanne A. Thum S. (2018). Circulating microRNA-132 Levels Improve Risk Prediction for Heart Failure Hospitalization in Patients with Chronic Heart Failure. Eur. J. Heart Fail. 20 (1), 78–85. 10.1002/ejhf.961 29027324

[B68] Moghiman T. Barghchi B. Esmaeili S. A. Shabestari M. M. Tabaee S. S. Momtazi-Borojeni A. A. (2021). Therapeutic Angiogenesis with Exosomal microRNAs: an Effectual Approach for the Treatment of Myocardial Ischemia. Heart Fail. Rev. 26 (1), 205–213. 10.1007/s10741-020-10001-9 32632768

[B69] Narasimhan G. Carrillo E. D. Hernández A. García M. C. Sánchez J. A. (2018). Protective Action of Diazoxide on Isoproterenol-Induced Hypertrophy Is Mediated by Reduction in MicroRNA-132 Expression. J. Cardiovasc. Pharmacol. 72 (5), 222–230. 10.1097/FJC.0000000000000619 30403388

[B70] Ni Y. G. Berenji K. Wang N. Oh M. Sachan N. Dey A. (2006). Foxo Transcription Factors blunt Cardiac Hypertrophy by Inhibiting Calcineurin Signaling. Circulation 114 (11), 1159–1168. 10.1161/CIRCULATIONAHA.106.637124 16952979 PMC4118290

[B71] Nicholls M. (2021). Recognition for Heart Failure Breakthrough. Eur. Heart J. ehab321. 10.1093/eurheartj/ehab321 Online ahead of print 34115838

[B72] Panico C. Condorelli G. (2018). microRNA-132: A New Biomarker of Heart Failure at Last? Eur. J. Heart Fail. 20, 86–88. 10.1002/ejhf.1044 29052296

[B73] Qiao G. Xia D. Cheng Z. Zhang G. (2017). miR132 in Atrial Fibrillation Directly Targets Connective Tissue Growth Factor. Mol. Med. Rep. 16 (4), 4143–4150. 10.3892/mmr.2017.7045 28731126

[B74] Rana I. Velkoska E. Patel S. K. Burrell L. M. Charchar F. J. (2015). MicroRNAs Mediate the Cardioprotective Effect of Angiotensin-Converting Enzyme Inhibition in Acute Kidney Injury. Am. J. Physiol. Ren. Physiol. 309 (11), F943–F954. 10.1152/ajprenal.00183.2015 26400542

[B75] Rawal S. Munasinghe P. E. Shindikar A. Paulin J. Cameron V. Manning P. (2017). Down-regulation of Proangiogenic microRNA-126 and microRNA-132 are Early Modulators of Diabetic Cardiac Microangiopathy. Cardiovasc. Res. 113 (1), 90–101. 10.1093/cvr/cvw235 28065883

[B76] Remenyi J. Hunter C. J. Cole C. Ando H. Impey S. Monk C. E. (2010). Regulation of the miR-212/132 Locus by MSK1 and CREB in Response to Neurotrophins. Biochem. J. 428 (2), 281–291. 10.1042/BJ20100024 20307261

[B77] Robson A. (2021). Inhibition of miR-132 Prevents the Progression of Heart Failure. Nat. Rev. Cardiol. 18, 612. 10.1038/s41569-021-00592-7 34155375

[B78] Ronnebaum S. M. Patterson C. (2010). The FoxO Family in Cardiac Function and Dysfunction. Annu. Rev. Physiol. 72, 81–94. 10.1146/annurev-physiol-021909-135931 20148668 PMC2908381

[B79] Ruan H. Li J. Ren S. Gao J. Li G. Kim R. (2009). Inducible and Cardiac Specific PTEN Inactivation Protects Ischemia/reperfusion Injury. J. Mol. Cel Cardiol. 46 (2), 193–200. 10.1016/j.yjmcc.2008.10.021 19038262

[B80] Šatrauskienė A. Navickas R. Laucevičius A. Krilavičius T. Užupytė R. Zdanytė M. (2021). Mir-1, miR-122, miR-132, and miR-133 are Related to Subclinical Aortic Atherosclerosis Associated with Metabolic Syndrome. Int. J. Environ. Res. Public Health 18 (4), 1483. 10.3390/ijerph18041483 33557426 PMC7915826

[B81] Schimmel K. Stojanović S. D. Huang C. K. Jung M. Meyer M. H. Xiao K. (2021). Combined High-Throughput Library Screening and Next Generation RNA Sequencing Uncover microRNAs Controlling Human Cardiac Fibroblast Biology. J. Mol. Cell Cardiol. 150, 91–100. 10.1016/j.yjmcc.2020.10.008 33127387

[B82] Sengupta A. Molkentin J. D. Yutzey K. E. (2009). FoxO Transcription Factors Promote Autophagy in Cardiomyocytes. J. Biol. Chem. 284 (41), 28319–28331. 10.1074/jbc.M109.024406 19696026 PMC2788882

[B83] Su Q. Liu Y. Lv X. W. Dai R. X. Yang X. H. Kong B. H. (2020). LncRNA TUG1 Mediates Ischemic Myocardial Injury by Targeting miR-132-3p/HDAC3 axis. Am. J. Physiol. Heart Circ. Physiol. 318 (2), H332–h344. 10.1152/ajpheart.00444.2019 31858814

[B84] Täubel J. Hauke W. Rump S. Viereck J. Batkai S. Poetzsch J. (2021). Novel Antisense Therapy Targeting microRNA-132 in Patients with Heart Failure: Results of a First-In-Human Phase 1b Randomized, Double-Blind, Placebo-Controlled Study. Eur. Heart J. 42 (2), 178–188. 10.1093/eurheartj/ehaa898 33245749 PMC7954267

[B85] Thum T. Galuppo P. Wolf C. Fiedler J. Kneitz S. van Laake L. W. (2007). MicroRNAs in the Human Heart: A Clue to Fetal Gene Reprogramming in Heart Failure. Circulation 116 (3), 258–267. 10.1161/CIRCULATIONAHA.107.687947 17606841

[B86] Tognini P. Pizzorusso T. (2012). MicroRNA212/132 Family: Molecular Transducer of Neuronal Function and Plasticity. Int. J. Biochem. Cel Biol. 44 (1), 6–10. 10.1016/j.biocel.2011.10.015 22062950

[B87] Ucar A. Gupta S. K. Fiedler J. Erikci E. Kardasinski M. Batkai S. (2012). The miRNA-212/132 Family Regulates Both Cardiac Hypertrophy and Cardiomyocyte Autophagy. Nat. Commun. 3 (1), 1078–1111. 10.1038/ncomms2090 23011132 PMC3657998

[B88] van Rooij E. Kauppinen S. (2014). Development of microRNA Therapeutics Is Coming of Age. EMBO Mol. Med. 6 (7), 851–864. 10.15252/emmm.201100899 24935956 PMC4119351

[B89] van Rooij E. (2011). The Art of microRNA Research. Circ. Res. 108 (2), 219–234. 10.1161/CIRCRESAHA.110.227496 21252150

[B90] Virani S. S. Alonso A. Benjamin E. J. Bittencourt M. S. Callaway C. W. Carson A. P. (2020). Heart Disease and Stroke Statistics-2020 Update: A Report From the American Heart Association. Circulation 141 (9), e139–e596. 10.1161/CIR.0000000000000757 31992061

[B91] Vo N. Klein M. E. Varlamova O. Keller D. M. Yamamoto T. Goodman R. H. (2005). A cAMP-Response Element Binding Protein-Induced microRNA Regulates Neuronal Morphogenesis. Proc. Natl. Acad. Sci. U S A. 102 (45), 16426–16431. 10.1073/pnas.0508448102 16260724 PMC1283476

[B92] Wahlquist C. Jeong D. Rojas-Muñoz A. Kho C. Lee A. Mitsuyama S. (2014). Inhibition of miR-25 Improves Cardiac Contractility in the Failing Heart. Nature 508 (7497), 531–535. 10.1038/nature13073 24670661 PMC4131725

[B93] Wanet A. Tacheny A. Arnould T. Renard P. (2012). miR-212/132 Expression and Functions: within and beyond the Neuronal Compartment. Nucleic Acids Res. 40 (11), 4742–4753. 10.1093/nar/gks151 22362752 PMC3367188

[B94] Wang G. Wang R. Ruan Z. Liu L. Li Y. Zhu L. (2020). MicroRNA-132 Attenuated Cardiac Fibrosis in Myocardial Infarction-Induced Heart Failure Rats. Biosci. Rep. 40 (9), BSR20201696. 10.1042/BSR20201696 32885809 PMC7494995

[B95] Xiong M. Jia C. Cui J. Wang P. Du X. Yang Q. (2015). Shexiang Tongxin Dropping Pill Attenuates Atherosclerotic Lesions in ApoE Deficient Mouse Model. J. Ethnopharmacol. 159, 84–92. 10.1016/j.jep.2014.11.013 25449459

[B96] Xu Q. Liang Y. Liu X. Zhang C. Liu X. Li H. (2019). miR132 Inhibits High Glucose Induced Vascular Smooth Muscle Cell Proliferation and Migration by Targeting E2F5. Mol. Med. Rep. 20 (2), 2012–2020. 10.3892/mmr.2019.10380 31257477

[B97] Zeller T. Keller T. Ojeda F. Reichlin T. Twerenbold R. Tzikas S. (2014). Assessment of microRNAs in Patients with Unstable Angina Pectoris. Eur. Heart J. 35 (31), 2106–2114. 10.1093/eurheartj/ehu151 24727883

[B98] Zhang C. J. Huang Y. Lu J. D. Lin J. Ge Z. R. Huang H. (2019). Retracted : Upregulated microRNA‐132 Rescues Cardiac Fibrosis and Restores Cardiocyte Proliferation in Dilated Cardiomyopathy through the Phosphatase and Tensin Homolog-Mediated PI3K/Akt Signal Transduction Pathway. J. Cell Biochem. 120 (2), 1232–1244. 10.1002/jcb.27081 30216493

[B99] Zhang J. Xu H. Gong L. Liu L. (2020). MicroRNA-132 Protects H9c2 Cells Against Oxygen and Glucose Deprivation-Evoked Injury by Targeting FOXO3A. J. Cell Physiol. 235 (1), 176–184. 10.1002/jcp.28956 31210352

[B100] Zhang L. Huang D. Wang Q. Shen D. Wang Y. Chen B. (2014). MiR-132 Inhibits Expression of SIRT1 and Induces Pro-inflammatory Processes of Vascular Endothelial Inflammation through Blockade of the SREBP-1c Metabolic Pathway. Cardiovasc. Drugs Ther. 28 (4), 303–311. 10.1007/s10557-014-6533-x 24924687

[B101] Zhao Z. Du S. Shen S. Wang L. (2020). microRNA-132 Inhibits Cardiomyocyte Apoptosis and Myocardial Remodeling in Myocardial Infarction by Targeting IL-1β. J. Cell Physiol. 235 (3), 2710–2721. 10.1002/jcp.29175 31621911

[B102] Zhou Y. Li K. S. Liu L. Li S. L. (2020). MicroRNA132 Promotes Oxidative Stress Induced Pyroptosis by Targeting Sirtuin 1 in Myocardial Ischaemia Reperfusion Injury. Int. J. Mol. Med. 45 (6), 1942–1950. 10.3892/ijmm.2020.4557 32236570

